# Integration of mult-omics and nucleotide metabolism reprogramming signature analysis reveals gastric cancer immunological and prognostic features

**DOI:** 10.1186/s12935-024-03396-0

**Published:** 2024-06-16

**Authors:** Shaofei Chen, Zhiyong Wang

**Affiliations:** Gastrointestinal Surgery, Wuhan Union Hospital, Wuhan, 430022 Hubei China

**Keywords:** Gastric cancer, Nucleotide metabolism, Prognosis, Immunotherapy efficacy, SERPINE1

## Abstract

**Background:**

Gastric cancer is a frequent and lethal solid tumor that has a poor prognosis and treatment result. Reprogramming of nucleotide metabolism is a characteristic of cancer development and progression.

**Methods:**

We used a variety of machine learning techniques to create a novel nucleotide metabolism-related index (NMRI) using gastric cancer sample data obtained from the TCGA and GEO databases. This index is based on genes associated to nucleotide metabolism. Gastric cancer patients were categorized into high and low NMRI groups based on NMRI results. The clinical features, tumor immune microenvironment, response to chemotherapy, and response to immunotherapy were then thoroughly examined. In vitro experiments were then used to confirm the biological role of SERPINE1 in gastric cancer.

**Results:**

The four nucleotide metabolism-related genes that make up NMRI (GAMT, ORC1, CNGB3, and SERPINE1) were verified in an external dataset and are a valid predictor of prognosis for patients with gastric cancer. The high NMRI group was more responsive to immunotherapy and had greater levels of immune cell infiltration than the low NMRI group. The proliferation and migration of stomach cancer was shown to be decreased by SERPINE1 knockdown in vitro.

**Conclusions:**

This study's NMRI can reliably predict a patient's prognosis for stomach cancer and pinpoint the patient group that will benefit from immunotherapy, offering important new information on the clinical treatment of stomach cancer.

**Supplementary Information:**

The online version contains supplementary material available at 10.1186/s12935-024-03396-0.

## Background

The second most prevalent cancer killer globally, gastric cancer (GC) is a malignant tumor that often affects the digestive tract [1, 2]. Gastric cancer risk factors include a history of *Helicobacter pylori* infection, a family history of the disease, and a diet high in nitrates and nitrites [3]. Current treatments have made great strides in slowing the growth of gastric cancer, but patients with advanced gastric cancer still have poor long-term survival rates [4]. According to recent research, immunotherapy can extend a patient's survival time if they have advanced stomach cancer, however this is not always the case for these individuals [5]. As a result, developing novel strategies for clinical first-line therapy for patients with stomach cancer is critically needed. Changes in metabolism are a fundamental characteristic of cancer cells, and increased nucleotide production and utilization are essential and common in all forms of cancer cells [6]. Nucleotide metabolic reprogramming plays a major role in the course of cancer, including unchecked proliferation, resistance to apoptosis, prolonged angiogenesis, immunological evasion, and metastasis [7].

It has been demonstrated that nucleotide metabolism is crucial for the development of tumors and the control of the immunological milieu [8, 9]. Nevertheless, a thorough synopsis of the connection between nucleotide metabolism and stomach cancer is still lacking. In order to predict the prognosis of patients with gastric cancer and the efficacy of treatment interventions, we gathered nucleotide metabolism-related genes in this study and used machine learning techniques to create a new indicator, the nucleotide metabolism-related index (NMRI). Finally, using in vitro tests, we assessed SERPINE1's involvement in gastric cancer.

## Materials and methods

### Data source

We extracted transcriptomic data and clinical data data of gastric cancer patients from the The Cancer Genome Atlas (TCGA) database (https://portal.gdc.cancer.gov/) [10]. From the GeneCards database and review publications, we gathered expressed genes linked to nucleotide metabolism as NM-related genes (Table S1). Furthermore, as a validation cohort, expression and clinical data related to stomach cancer were retrieved from the GEO database (ID: GSE84437). All gene sets were processed by homogenization. The Human Protein Atlas database (https://www.proteinatlas.org/) [11] was also searched to obtain histological validation of GAMT, ORC1, CNGB3, and SERPINE1 at the protein level between gastric cancer tumor tissues and gastric normal tissues.

### Construction and validation of nucleotide metabolism-related index (NMRI)

Following differential (LogFc > 1; fdr < 0.05) and prognostic (p < 0.05) analyses of the expression data from the training dataset (TCGA samples), the four NM-related genes were identified by univariate and multivariate Cox regression analyses and built as NMRI. The following formula was used to determine each stomach cancer sample's NMRI score: Coef(Gene 1) × Expr(Gene 1) + Coef(Gene 2) × Expr(Gene 2) + …… + Coef(Gene n) × Expr(Gene n) is the NMRI (univariate cox regression analysis was used to screen for prognostically relevant genes, and multivariate cox regression analysis allowed simultaneous analysis of the effects of multiple predictors on the occurrence of survival events.). Expr(Gene) denotes the expression of Gene and Coef(Gene) denotes the risk regression coefficient of Gene. We divided gastric cancer patients into two groups: patients with high NMRI and patients with low NMRI, based on the median value of NMRI.

### Unsupervised clustering of NMRI-related genes

In order to discover nucleotide metabolism-associated gastric cancer subtypes, we used consensus clustering using the R package "ConsensusClusterPlus" based on the intersection of NMRI-associated genes and gastric cancer and normal tissue differential genes [12].

### Immune microenvironment analysis

Tumor purity, ESTIMATE score, immune cell score, and stroma score were computed for every sample using the R package "ESTIMATE" [13]. The relative percentage of immune cell infiltration was measured using the Single Sample Gene Set Enrichment Analysis (ssGSEA) technique. Additionally, seven software systems, including XCELL, carried out immune cell correlation analysis.

### Chemotherapy response and immunotherapy response

By using the ProPhetic method to determine the IC50 values for various chemotherapeutic and targeted therapeutic medicines, we were able to evaluate the responsiveness of patients to medication. Patients' responses to immunotherapy can be predicted using the Tumor Immune Dysfunction and Exclusion (TIDE) algorithm; higher TIDE scores are frequently linked to worse treatment responses and more immune escape potential [14]. Additionally, we used the TCIA database (https://tcia.at/home) to download IPS score data for gastric cancer in order to evaluate patient response to immune checkpoint inhibitors such as anti-PD-1 and anti-CTLA4 [15]. The Real World Immunotherapy dataset (Imvigor210 dataset) was used to validate immunotherapy effects.

### Molecular dock

Using Schrödinger software, we screened and ran molecular docking simulations. The PDB database was used to obtain the target target's protein structure (SERPINE1-7AQG), and the PubChem database (https://pubchem.ncbi.nlm.nih.gov/) was used to obtain the structure of the natural small molecule medication. The molecular docking module of Schrödinger program was utilized to mimic the binding positions of SERPINE1 with small molecule medicines.

### Small interfering RNA (siRNA) transfection

MKN45 and HGC27 cells were inoculated in cell culture plates and SERPINE1 was knocked down using small interfering RNA (siRNA) according to the kit instructions.

### CCK8 experiment

MKN45 and HGC27 cells were spread into 96-well plates, and 100 μL of CCK-8 solution was added to each well at 24, 48, 72, and 96 h. The plates were then incubated in an incubator, and the absorbance values of each well were determined using an enzyme meter 2 h later.

### Wound-healing and transwell experiments

The next day, MKN45 and HGC27 cells were resuspended and arranged on the plate. Next, scratches were made with a straightedge, and the floating cells were removed and examined under a microscope after 48 h. Serum-free media was used to starve transfected MKN45 and HGC27 cells for a duration of 4 h. Trypsin was used to break down the cells and resuscitate them in incomplete media without serum. The cells were then incubated for 48 h in two different halves of the Transwell: the top chamber, which contained the cell suspension, and the lower chamber, which contained complete medium with fetal bovine serum. After cleaning the Transwell, the cells were stained with crystalline violet and preserved with 4% paraformaldehyde. After being cleaned and preserved with 4% paraformaldehyde, the cells were stained with crystal violet.

### Statistical analysis

The "limma" R program was used to assess the differences between the surrounding normal tissue and stomach cancer. The Kaplan–Meier technique was utilized to plot survival curves and examine the variations in survival between the two groups. Spearman correlation analysis was used to evaluate correlation. A p-value of less than 0.05 was deemed statistically significant. R conducted all statistical analyses.

## Results

### Identification of nucleotide metabolism-related clusters and differences in immune microenvironment and immunotherapeutic response among different clusters

**Fig. 1:**
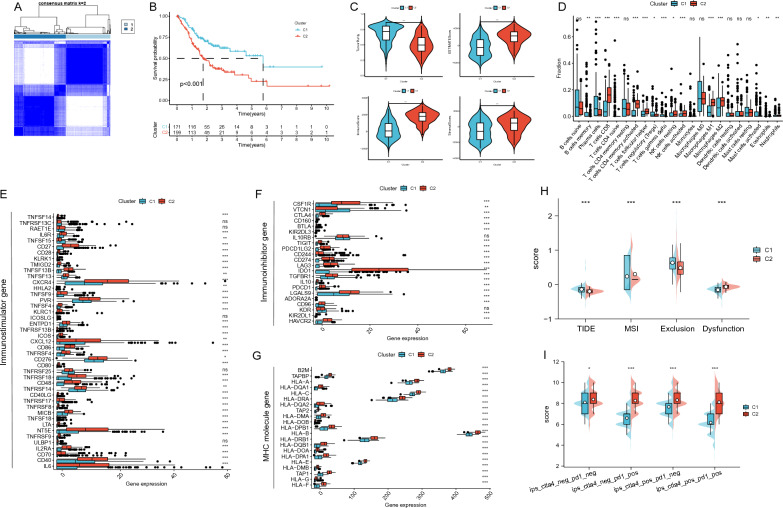
Nucleotide metabolism clustering analysis and association between different Clusters with the immune microenvironment. **A** Heatmap of consensus clustering at k = 2. **B** Comparison of Kaplan–Meier curves between Cluster 1 and Cluster 2. **C** Comparison of ESITIMATE scores for tumor purity, stroma and immunity between two Clusters. **D** Comparison of CIBERSORT score for immune cell infiltration between the two Clusters. Comparison of the expression of common immunostimulatory genes (**E**), immunosuppressive genes (**F**) and MHC molecular genes (**G**) between the two Clusters. **H** Comparison of TIDE, MSI, Exclusion and Dysfunction between the two Clusters. **I** Differences in response to no PD1 or CTLA4 blocker, PD1 blocker, CTLA4 blocker and PD1-CTLA4 co-blocker between the two Clusters. Note * p < 0.05, **p < 0.01, ***p < 0.001

Using nucleotide metabolism-related genes (Table S1) gathered from the GeneCards database and review articles, we conducted cluster analysis on gastric cancer patients. The results demonstrated that the patients could be well classified into two Clusters, with the two Clusters having superior internal stability and consistency (Fig. 1A). According to the Kaplan–Meier curves (Fig. 1B), patients in the Cluster 1 group had a considerably better prognosis than those in the Cluster 2 group (p < 0.05). Compared to the Cluster2 group, they had a considerably better prognosis (p < 0.05) (Fig. 1B). When the immune microenvironment differences between the two Clusters were examined using the ESITIMATE method, Cluster 2 outperformed Cluster 1 in terms of immune scores, stromal scores, ESITIMATE scores, and tumor purity (Fig. 1C). According to Fig. 1D, the CIBERSORT algorithm revealed a substantial difference in immune cell infiltration between the two clusters, with the weight of CD8 T cells in Cluster 2 being considerably larger than in Cluster 1 (p < 0.05). The majority of the immunostimulatory genes, immunosuppressive genes, and MHC molecules were found to be significantly more expressed in Cluster 2 than in Cluster 1 when we also compared the expression of common immunostimulatory, immunosuppressive, and MHC molecule genes between the two Clusters (Fig. 1E–G). Additionally, we discovered that, compared to Cluster 1, Cluster 2 had much greater TIDE score and dysfunction, whereas Cluster 1 had significantly higher MSI and exclusion (Fig. 1H). Lastly, we examined the IPS scores against the effects of anti-PD-1 and anti-CTLA-4 treatment. The results indicated that the scores for CTLA-4(-)PD-1( +), CTLA-4( +)PD-1(−), and CTLA-4( +)PD-1( +) were significantly higher in Cluster 2 than in Cluster 1, indicating that the effects of anti-CTLA-4 or anti-PDLA-4 treatment were significantly higher in Cluster 2 than in Cluster 1, indicating that the effects of anti-CTLA-4 or anti-PD-1 treatment were significantly lower in Cluster 2 than in Cluster 1. The more successful immune checkpoint inhibitors were CTLA4 or anti-PD-1 (Fig. 1I).

### Construction and validation of NMRI

**Fig. 2:**
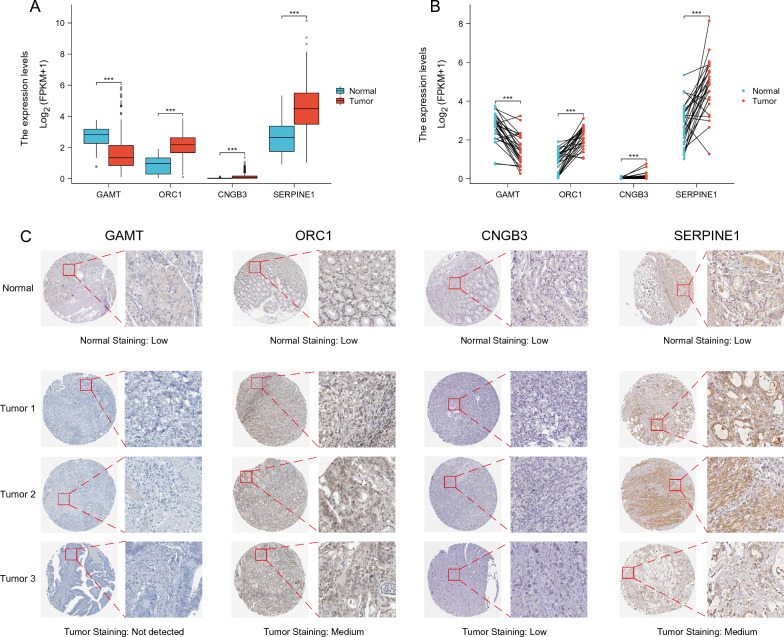
Construction of nucleotide metabolism-related indices and validation of immunohistochemical staining results. **A** Differences in the expression levels of GAMT, ORC1, CNGB3 and SERPINE1 in unpaired gastric cancer tissues and normal gastric tissues in TCGA database. **B** Differences in the expression levels of GAMT, ORC1, CNGB3 and SERPINE1 in paired gastric cancer tissues and normal gastric tissues in the TCGA database. **C** Immunohistochemical staining results of GAMT, ORC1, CNGB3 and SERPINE1 in normal gastric tissues and gastric cancer tissues. Note * p < 0.05, **p < 0.01, ***p < 0.001

We collected expressed nucleotide metabolism-related genes (Table S1) from the GeneCards database and review articles for variance analysis (LogFc > 1; fdr < 0.05) and prognostic analysis (p < 0.05), after which, in order to construct the nucleotide metabolism-related risk model and its derived Nucleotide Metabolism-Related Index (NMRI), we screened out, by using one-way Cox analysis, the four genes with independent prognostic value to construct NMRI, which were GAMT (HR = 1.058, 95% CI 1.021–1.096, p = 0.002), ORC1 (HR = 0.896, 95% CI 0.810–0.991, p = 0.033), CNGB3 (HR = 2.800, 95% CI 1.137–6.896, P = 0.025) and SERPINE1 (HR = 1.032, 95% CI 1.017–1.047, P = 0.001).The coefficients obtained from multifactorial Cox analysis of the four genes in the NMRI, the GAMT coefficient was 0.051, the ORC1 coefficient was -0.094, the CNGB3 coefficient was 1.234 and the coefficient of SERPINE1 was 0.030. After that, we compared the differences in the expression levels of GAMT, ORC1, CNGB3, and SERPINE1 in unpaired and paired gastric cancer tissues and normal gastric tissues from the TCGA database, and the results showed that the expression of the GAMT gene was significantly down-regulated in the gastric cancer tissues, and that the expression of ORC1, CNGB3, and SERPINE1 was significantly up-regulated in the tumor tissues with significantly up-regulated expression (Fig. 2A−B). We also obtained immunohistochemical staining results of GAMT, ORC1, CNGB3 and SERPINE1 in normal gastric tissues and gastric cancer tissues from the HPA database showed that GAMT was decreased in IHC staining in gastric cancer tissues, whereas ORC1, CNGB3 and SERPINE1 were all increased in IHC staining in tumor tissues (Fig. 2C).

## Nucleotide metabolism-related index predicts prognosis in gastric cancer patients

**Fig. 3:**
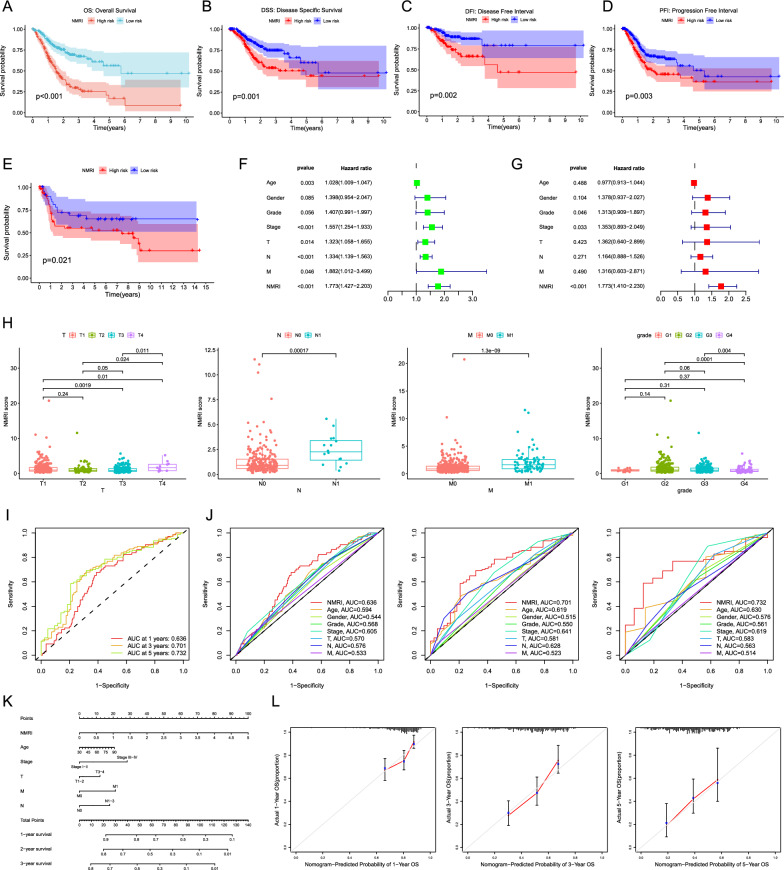
Association of nucleotide metabolism-related indices with clinical traits and constructed column line graphs. survival curves of the TCGA cohort for overall survival (OS) (**A**), disease-specific survival (DSS) (**B**), disease-free interval (DFI) (**C**) and progression-free interval (PFI) (**D**). **E **Survival curves for the GSE84437 cohort. **F-G** Univariate and multivariate regression analysis. **H** Differences in NMRI scores between different clinical features (T, M, N and grading). **I** NMRI ROC curves at 1, 3 and 5 years. **J** AUC comparisons of 1-, 3-, and 5-year NMRI with other clinical traits. **K **Column line graphs on NMRI constructs. **L** Calibration curves for 1-, 3-, and 5-year column line plots. Note * p < 0.05, **p < 0.01, ***p < 0.001

We first analyzed NMRI to predict the prognosis of gastric cancer patients by comparing the differences in survival between high and low NMRI in overall survival (OS), disease-specific survival (DSS), disease-free interval (DFI), and progression-free interval (PFI), respectively, and the results of the survival curves showed that, compared with low NMRI, the high NMRI group had a OS, DSS, DFI, and PFI poorer prognosis (p < 0.05) (Fig. 3A−D). Using the GSE84437 cohort as a validation cohort, we assessed the prognostic predictive validity of NMRI. The findings also indicated that patients in the high NMRI group had a significantly worse prognosis than those in the low NMRI group (p < 0.05), indicating that NMRI may be a more reliable predictor of patient prognosis for gastric cancer (Fig. 3E). NMRI was found to be a risk factor regardless of other clinical features based on the outcomes of univariate and multivariate regression analysis (Fig. 3F−G). Additionally, we calculated the differences in NMRI scores for other common clinical features. The results indicated substantial differences (p < 0.05) in T, N, M, and grade differences (Fig. 3H).

The validity of NMRI prognostic prediction was assessed using the area under the ROC curve (AUC). The 1-year, 3-year, and 5-year AUCs of NMRI prognostic prediction were 0.636, 0.701, and 0.732, respectively. The AUC values of 1-year, 3-year, and 5-year were superior to the other clinical traits for predicting the survival status of patients, indicating that NMRI is a more accurate predictor of the survival status of both short- and long-term gastric cancer patients (Fig. 3I–J). Ultimately, we created column line plots utilizing NMRI and additional clinical characteristics (age, clinical stage, T, N, and M), with NMRI accounting for the majority of the column line plots' overall score (Fig. 3K). When compared to the reference line, the 1-, 3-, and 5-year column line plots demonstrated acceptable prediction accuracy, according to the calibration curves of the column line plots (Fig. 3L). These findings imply that NMRI is a valid and trustworthy method for predicting patients' chances of surviving stomach cancer.

### Gene set enrichment analysis and correlation study of NMRI with tumor microenvironment

To explore the cancer signature pathways associated with NMRI, we performed GSEA analysis in the high and low NMRI groups, which showed that the high NMRI group was significantly enriched in ANGIOGENESIS, EPITHELIAL MESENCHYMAL TRANSITION and HYPOXIA signaling pathways, and the low NMRI group was significantly enriched in the DNA REPAIR and OXIDATIVE PHOSPHORYLATION signaling pathways (Fig. 3B). In addition, GO enrichment analysis of the high NMRI group revealed that high NMRI was enriched on multiple immune cell infiltration signaling pathways, such as B CELL MEDIATED IMMUNITY, T CELL MEDIATED IMMUNITY, REGULATION OF B CELL ACTIVATION, REGULATION OF T CELL ACTIVATION and T CELL RECEPTOR COMPLEX signaling pathways (Fig. 3B).

**Fig. 4:**
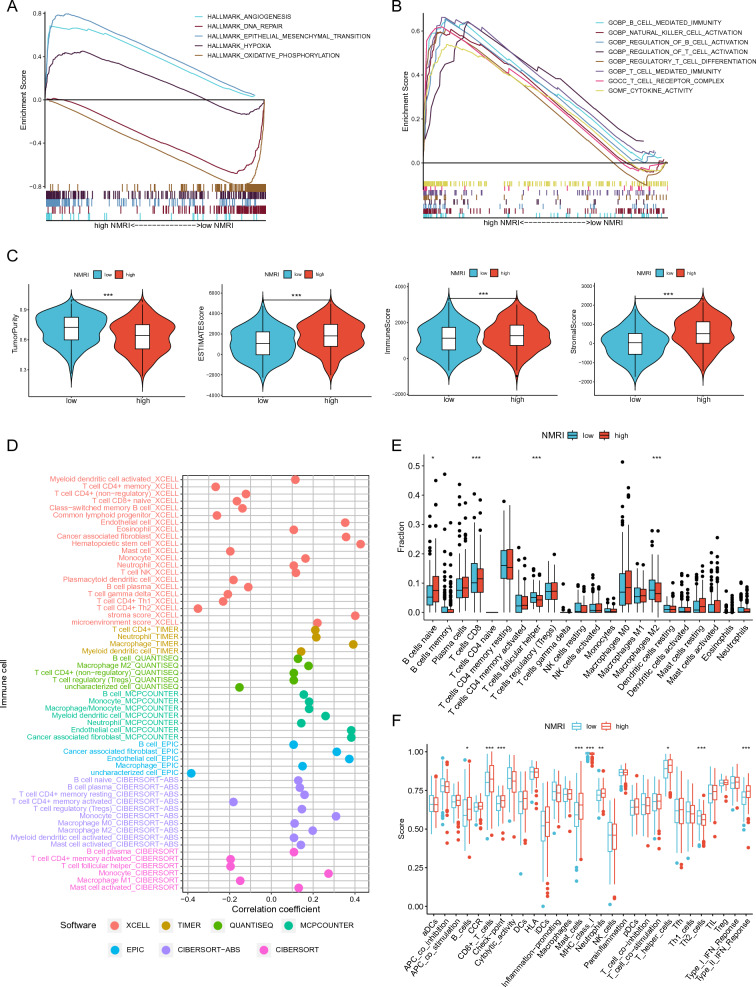
Correlation analysis of GSEA and tumor microenvironment. **A-B** GSEA analysis of patients in the high NMRI group. **C** Comparison of tumor purity, ESTIMATE score, immune score and stromal score of patients in the high/low NMRI group. **D** Seven software analyses of NMRI correlating with various immune cell infiltration levels. **E** The CIBERSORT algorithm compares the differences in immune cell infiltration levels between high/low NMRI groups. **F** The ssGSEA algorithm analyzes differences in immune cell infiltration and immune-related functions between high/low NMRI groups

The TME scores (ESTIMATE score, immune score, and stroma score) of the patients in the high-NMRI group were significantly higher than those in the low-NMRI group, while the tumor purity scores were significantly lower than those in the low-NMRI group, indicating that the high-NMRI group had a higher level of immune infiltration (Fig. 4C). We used the ESTIMATE algorithm to assess the immune cell infiltration of the tumor microenvironment in gastric cancer patients. Using seven software programs, including XCELL, we examined the relationship between NMRI and immune cell infiltration. The results showed that NMRI and the majority of immune cells had a positive connection (Fig. 4D). The CIBERSORT algorithm's results demonstrated that while the amount of immunosuppressive M2-type macrophages was much lower in the high NMRI group, the level of immunostimulated CD8 T cells was significantly greater in the high NMRI group than in the low NMRI group (Fig. 4E). Furthermore, patients in the high-NMRI group had higher immune cell infiltration and immune-related activities than those in the low-NMRI group, according to the results of the ssGSEA algorithm used to assess these data (Fig. 4F). Taken together, the findings imply that patients with high NMRI values could also have significant levels of immune infiltration in stomach cancer.

### Association of NMRI with immunotherapy efficacy and patient response

**Fig. 5:**
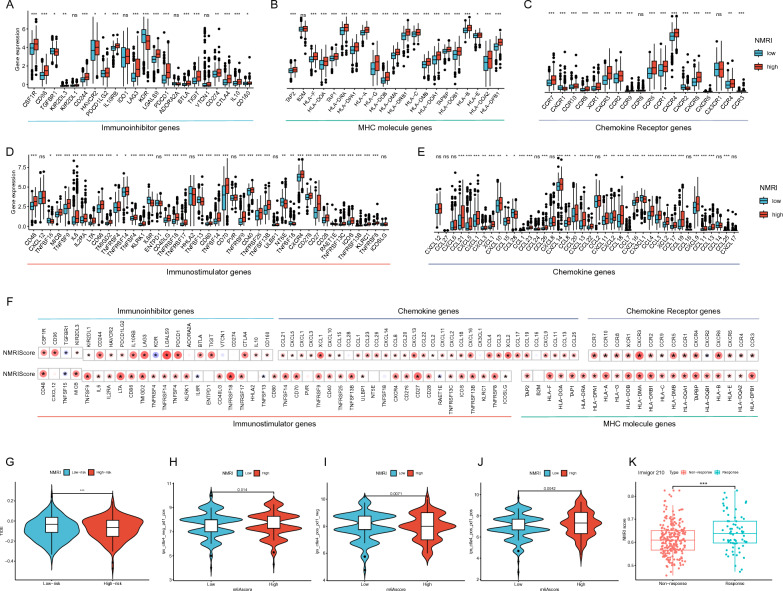
Application of NMRI in immunotherapy response. Differences in the expression levels of immunosuppressive genes (**A**), MHC molecules (**B**), cytokine receptors (**C**), immunostimulatory genes (**D**), and cytokines (**E**) between gastric cancer patients in high/low NMRI groups. **F** Heatmap of correlation between NMRI and immunostimulatory genes, immunosuppressive genes, MHC molecules, cytokines and cytokine receptors. **G** Differences in TIDE scores between gastric cancer patients in the high/low NMRI group. **H-J** Differences in IPS scores between gastric cancer patients in the high/low NMRI group. **K** External immunotherapy dataset Imvigor210 validates VMRI for immunotherapy effect prediction

We compared the expression levels of common immune checkpoints (immunosuppressive and immunostimulatory genes), MHC molecules, cytokines, and cytokine receptors between the high and low NMRI groups in order to investigate the relationship between NMRI and the immune microenvironment further. The results indicated that the expression levels of the majority of the aforementioned genes were significantly higher in the high NMRI group than in the low NMRI group (Fig. 5A−E). The association between NMRI and genes that stimulate the immune system, genes that repress the immune system, MHC molecules, cytokines, and cytokine receptors was next examined. The findings indicated that the majority of the genes had a substantial and positive connection with NMRI (p < 0.05, Fig. 5F). These findings imply that immunotherapy was more effectively received by patients in the high NMRI group.

According to the present study, patients with lower TIDE scores are more likely to benefit from immunotherapy, and TIDE and IPS scores can be used to evaluate a patient's response to immunotherapy [14]. The study revealed that the high NMRI group's TIDE scores were considerably higher than the low NMRI group's, indicating that the immune checkpoint inhibitor medication was more effective in the high NMRI group (Fig. 5G). Further investigation revealed that the immunophenotypic core (IPS) scores of the high NMRI group were significantly higher (p < 0.05) than those of the low NMRI group, indicating that the patients in the high NMRI group may be more responsive to immunotherapy (Fig. 5H−J). Additionally, we examined the relationship between the NMRI group and immunogenicity to predict patients' response to immune checkpoint blockade (anti-PD1 and/or anti-CTLA4). In order to verify the NMRI prediction of immunotherapy effect, we lastly gathered an external real immunotherapy dataset (Imvigor210). The outcomes demonstrated that the patients' NMRI scores in the group responding to anti-PD-L1 immunotherapy were significantly higher than those of the patients in the non-responding group (Fig. 5K). The findings demonstrated that NMRI may predict how well stomach cancer patients will respond to immunotherapy, with higher NMRI patients seeing better immunotherapy outcomes.

### NMRI correlation and molecular docking with common drug sensitivity

**Fig. 6:**
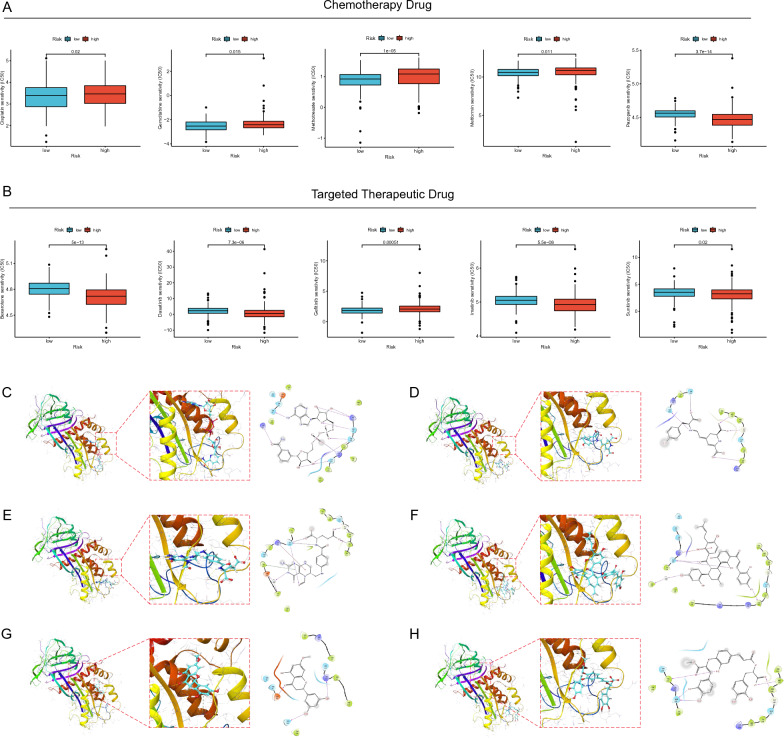
Application of NMRI in drug sensitivity and molecular docking. Differences in response to common gastric cancer chemotherapeutic drugs (**A**) and targeted therapeutic drugs (**B**) between high and low NMRI groups. The Figure shows the docking poses of the SERP INE1 activity pocket with Fenugreekine (**C**), P ortulacaxanthin II (**D**), Leucovorin (**E**), Kuwanon J (**F**), Blumeatin (**G**) and Schizotenuin F (**H**)

We examined the reactions of the high/low NMRI group to both standard gastric cancer chemotherapeutic treatments and targeted therapeutic pharmaceuticals in order to inform the clinical usage of medications in these patients. The IC50 of the drugs was shown to be inversely correlated with the patients' sensitivity to the drugs. The outcomes demonstrated that patients with low NMRI had better drug sensitivity, i.e., better therapeutic outcome, to Cisplatin, Gemcitabine, Methotrexate, Metformin, and Gefitinib, while patients with high NMRI had higher drug sensitivity to Pazopanib, Bexarotene, Dasatinib, Imatinib, and Sunitinib (Fig. 6A).

For chemical screening, a computer technique based on structure is called molecular docking. Using the PDB database, we were able to retrieve the protein structure of SERPINE1 (ID: 7AQG) for the purpose of molecular docking with small natural molecules. Figure 6C−H displays the top six small molecules (Fenugreekine, Portulacaxanthin II, Leucovorin, Kuwanon J, Blumeatin, and Schizotenuin F) that have the highest affinity for binding to the SERPINE1 binding pocket. As an illustration, Portulacaxanthin II forms hydrogen bonds with residues Gln-123, Thr-120, Met-110, Gly-108, and Leu-105 of the amino acid sequences of SERPINE1, where Gln-123 acts as an acceptor and Thr-120, Met-110, Gly-108, and Leu-105 as donors. Asp-96, His-143, and Arg-118 of the SERPINE1 amino acid residues establish hydrogen bonds with Blumeatin; Asp-96 is a hydrogen bond donor whereas Arg-118 and His-143 are hydrogen bond acceptors.

## Effects of SERPINE1 knockdown on proliferation and migration of gastric cancer cells

**Fig. 7:**
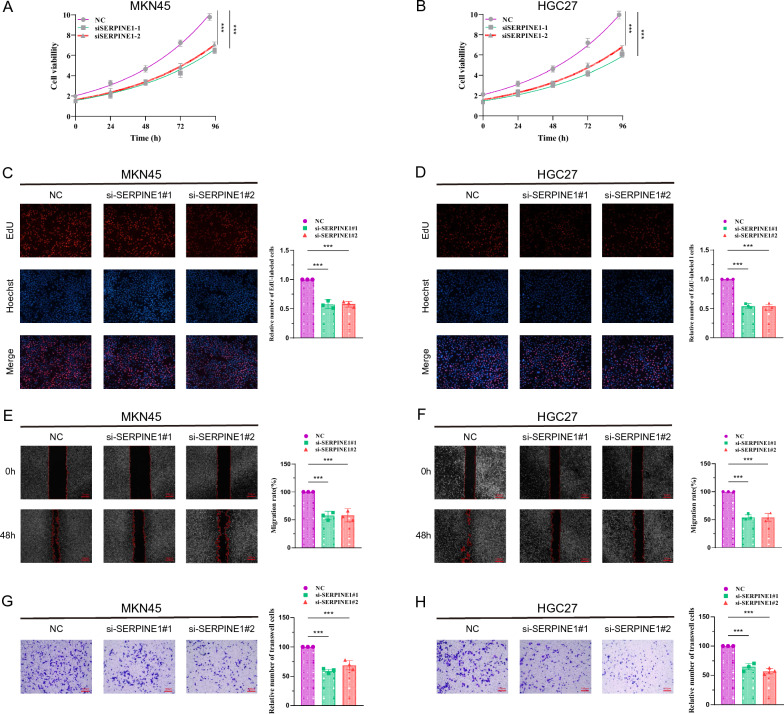
Knockdown of SERP INE1 inhibits gastric cancer cell proliferation and migration. CCK8 viability assay (**A**-**B**), EdU cell proliferation ability assay (**C-D**), wound healing ability assay (**E–F**) and Transwell cell migration ability (**G-H**) of MKN45 and HGC27 cells transfected with two si-SERPINE1, respectively. Note * p < 0.05, **p < 0.01, ***p < 0.001

We created two siRNAs (SERPINE1-1, SERPINE1-2) to mute SERPINE1 expression in MKN45 and HGC27 cells in order to study the function of SERPINE1 in gastric cancer cells. We transfected MKN45 and HGC27 cells with si-SERPINE1 (Figure S1 A-B), respectively, and used those cells for CCK8, EdU, wound healing, and Transwell experiments. The proliferative ability of MKN45 and HGC27 cells in both si-SERPINE1 groups was much less than that of the NC group at 24, 48, 72, and 96 h, according to cCK8 findings (Fig. 7A−B). The proliferation ability of MKN45 and HGC27 cells was much lower in the knockdown of the SERPINE1 gene than in the NC group, according to the findings of the EdU staining experiment (Fig. 7C−D). The findings of the Transwell test and the wound healing experiment showed a substantial reduction in the migratory capacity of MKN45 and HGC27 cells that had SERPINE1 knocked down (Fig. 7E–H). According to the aforementioned findings, reducing SERPINE1 expression may prevent stomach cancer cells from proliferating and migrating. In addition, we found that the proliferative capacity of MKN45 and HGC27 was significantly reduced by knocking down ORC1(Figure S1 C-D).

## Discussion

When stomach cancer is locally progressed or metastatic, it is an aggressive malignant tumor with a terrible prognosis [16]. Certain individuals with advanced gastric cancer have recently demonstrated success with immunocheckpoint inhibitors that target PD-1 or PD-L1 [3, 17]. Thus, in order to forecast the survival status and immunotherapy response in patients with gastric cancer, we require trustworthy markers. Using nucleotide metabolism-related genes derived from four nucleotide metabolism-related genes (GAMT, ORC1, CNGB3, and SERPINE1), we developed a nucleotide metabolism-related index (NMRI) in this work. We discovered that NMRI is able to predict not only the prognosis of patients with stomach cancer but also the effectiveness of immunotherapy in these patients, which might result in treatment approaches to enhance the prognosis of patients. Further investigation using in vitro tests and early virtual screening revealed SERPINE1's oncobiological role and possible druggability in gastric cancer. The derived nucleotide metabolism-related index can be used as a new indicator for predicting the prognosis of gastric cancer and the benefit of immunotherapy, and it may offer important insights into the hunt for novel therapeutic strategies for gastric cancer. As a result, the nucleotide metabolism-related model we constructed can predict the prognostic risk and immunotherapy response in gastric cancer.

This study's exploration of the oncological therapeutic potential of gastric cancer from the novel viewpoint of nucleotide metabolic reprogramming is a significant result. We compared the expression levels of common immune checkpoints and HLA molecules between the high and low NMRI groups to see if NMRI could predict the effectiveness of anticancer immunotherapy in gastric cancer patients. We discovered that the vast majority were significantly upregulated in the high NMRI group. It is possible that ICI immunotherapy is more successful in treating the high NMRI population since the TIDE scores in the high NMRI group were considerably lower than those in the low NMRI group. In keeping with these findings, the IMvigor 210 cohort's anti-PD-L1 treatment-responsive and non-responsive groups differed significantly in their NMRI scores, with the former having higher NMRI scores. On the other hand, using the IC50 values of both chemotherapeutic and targeted therapeutic drugs, we were able to anticipate how stomach cancer patients will react to these conventional treatments. These findings imply that our development of NMRI is a useful indicator for evaluating how well patients with gastric cancer respond to immunologic and pharmacological therapies. It can also precisely determine which patients are most likely to benefit from immunotherapy and provide a prognosis for gastric cancer patients.

The nucleotide metabolism-related index eventually contained four genes: GAMT, ORC1, CNGB3, and SERPINE1. The expression of GAMT was considerably down-regulated in gastric cancer tissues when compared to normal gastric tissues, while the expression of ORC1, CNGB3, and SERPINE1 was significantly up-regulated in gastric cancer tissues. Guanidinoacetate N-Methyltransferase (GAMT) encodes a methyltransferase that uses S-adenosylmethionine as a methyl donor to convert guanidinoacetate to creatine. This process causes creatine deficiency syndrome in the brain [18, 19]. The main component of the DNA replication complex pre-origin recognition complex, origin recognition complex subunit 1 (ORC1), can enhance DNA replication by enlisting the help of CDC6 and cell cycle protein E [20]. The beta subunit of the cyclic nucleotide-gated ion channel, which is involved in controlling channel function in cone photoreceptors, is encoded by the Cyclic Nucleotide Gated Channel Subunit Beta 3 (CNGB3) gene. Mutations in CNGB3 have been linked to a number of disorders, including color blindness and macular degeneration in teenagers [21, 22]. Serpin Family E Member 1 (SERPINE1) encodes a member of the serine protease inhibitor (serpin) superfamily, which plays an important role in the fibrinolytic system, and current studies have shown that SERPINE1 plays a role in the prognosis, drug resistance, and metastasis of a variety of tumors [23, 24]. According to the aforementioned findings, gastric cancer biological processes and tumor immunity are strongly linked to nucleotide metabolism-related genes (GAMT, ORC1, CNGB3, and SERPINE1) and their derived nucleotide metabolism-related indices (NMRIs).

We also showed the viability of a structure-based strategy to identify small molecule therapeutic candidates that can target core proteins. This demonstrates another application of NMRI for predicting therapeutic efficacy. In order to screen possible small-molecule medications by molecular docking using Schrödinger software, we employed SERPINE1 as a small-molecule drug target and downloaded natural small-molecule pharmaceuticals from the PubChem database. Leucovorin is one of the top six small molecule medications with the highest affinity to SERPINE1, and it is now utilized as a first-line therapy for advanced gastric and colorectal cancers together with fluorouracil and oxaliplatin [25, 26]. Blumeatin is isolated from the traditional Chinese medicine Blumea balsamifera, which has been demonstrated to have anti-inflammatory activity [27]. Although our molecular docking results indicate that these natural small molecules can bind to SERPINE1, the specific binding mechanism has yet to be deeply investigated.

## Conclusion

To summarize, our thorough examination of several facets of gastric cancer using NMRI, which is derived from genes associated with nucleotide metabolism, revealed that NMRI is a reliable tool for predicting a patient's prognosis and response to immunotherapy. From the standpoint of nucleotide metabolism, this study discovered novel combinations of prognostic and therapeutic biomarkers as well as possible therapeutic targets. These findings will be helpful for future studies on therapeutic approaches for gastric cancer. Investigating nucleotide metabolism reprogramming and its potential in cancer immunotherapy provides new insights for clinical diagnostics, personalized care, and translational research in gastric cancer.

### Supplementary Information

Additional file1.

## Data Availability

All data utilized in this study are included in this article and all data supporting the findings of this study are available on reasonable request from the corresponding author.

## Abbreviations

TCGA: The Cancer Genome Atlas
GEO: Gene Expression Omnibus
GTEx: Genotype-Tissue Expression Program
ROC: Receiver Operating Characteristic
PD-1: Programmed cell death 1
PD-L1: Programmed cell death 1 ligand 1
CTLA4: Cytotoxic T-lymphocyte-associated protein 4
ICB: Immune checkpoint blockade
OS: Overall survival
ROC: Receiver Operation Characteristic
NES: Normalized enrichment score
TMB: Tumor mutation burden
IC50: Half maximal inhibitory concentration
GDSC: Genomics of Drug Sensitivity in Cancer
PPI: Protein–protein interaction
KM: Kaplan–Meier
ssGSEA: Single-sample gene set enrichment analysis
TIDE: Tumor immune dysfunction and exclusion
GSEA: Gene set enrichment analysis
GO: Gene Ontology
KEGG: Kyoto Encyclopedia of Genes and Genomes
ORC1: Origin recognition complex subunit 1
GAMT: Guanidinoacetate N-Methyltransferase
CNGB3: Cyclic Nucleotide Gated Channel Subunit Beta 3
SERPINE1: Serpin Family E Member 1
