# Supplementary material for: Integration of mult-omics and nucleotide metabolism reprogramming signature analysis reveals gastric cancer immunological and prognostic features
Source: Cancer Cell Int. 2024 Jun 16;24:212. doi: 10.1186/s12935-024-03396-0 (PMC11180389; doi:10.1186/s12935-024-03396-0)
Supplement: Supplementary file 1 — Additional file1. [file 12935_2024_3396_MOESM1_ESM.docx]

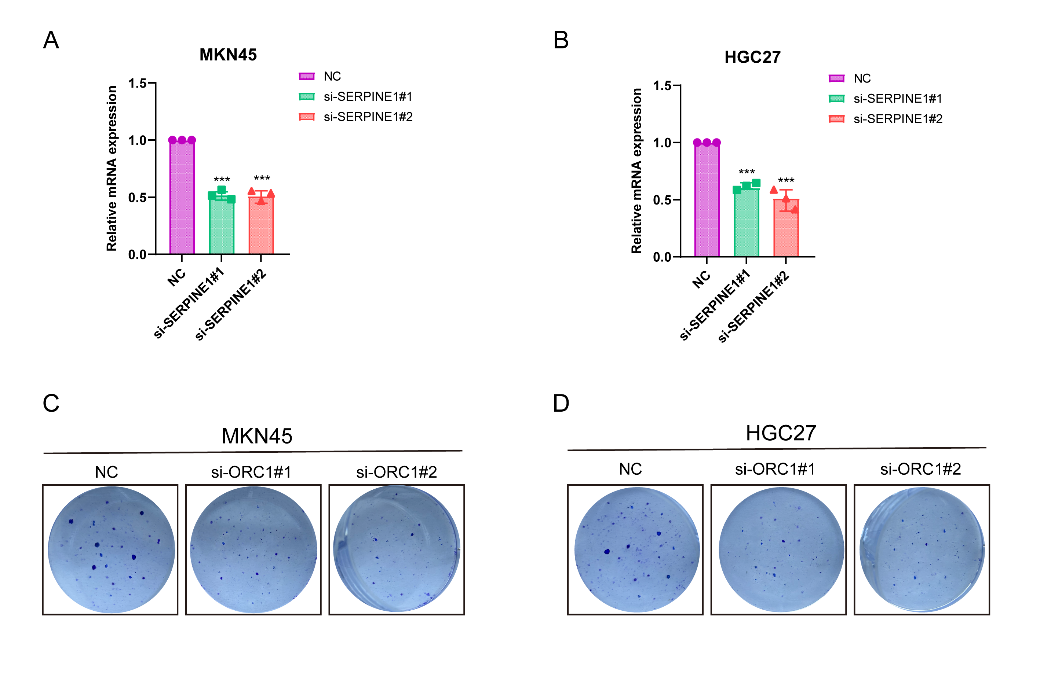


**Figure S1 （A-B）QPCR validation of SERPINE1 mRNA expression levels in MKN45 and HGC27 cell after transfection with siRNA.（C-D）Plate cloning of MKN45 and HGC27 cells transfected with two si-ORC1.**

**TableS1 Nucleotide metabolism gene**

ALG3

SEMA4B

CENPF

PLK4

RFPL3S

MAS1L

ADSL

E2F1

NUP205

METAP1

AHCYL2

NUP50

CABYR

GPSM2

SAC3D1

DONSON

HDAC2

HLCS

HPRT1

ILF3

KCNN4

ACLY

NFYA

NME1

NME2

NUP88

ORC1

GMNN

UTP18

ZNF706

MRTO4

RAB6B

PADI3

PMCHL1

POLA1

SLC30A10

PLGRKT

SLC25A37

PRPS2

MDM1

ZNF490

RRM1

RRM2

ANKEF1

SNRPA

SRPK1

BYSL

UCK2

ZIC2

ALMS1

IFRD2

SPEF2

CDC45

PLEKHA8

PRPF38A

DLEU2

IER3

DDX21

NUP155

MDC1

CD70

ARHGAP11A

NCAPD2

ZBTB5

MTR

CYP2D6

MMACHC

ABCA3

CYP2C19

SLC19A3

APOE

CYP3A4

MTRR

INS

MTHFR

TPK1

ADIPOQ

MMADHC

ACADM

PPARG

LDLR

CETP

CYP2C9

IL6

LPL

TPMT

SFTPB

LEP

CYP1A2

LIPC

CSF2RA

SFTPC

ERCC2

TNF

CYP2B6

APOB

CYP1A1

CYP2A6

ENPP1

CYP2E1

TP53

CYP3A5

NUDT15

CYP2C8

SLC25A4

DPYD

CBS

PPARA

MMAA

VDR

ERCC1

ABCG5

OTC

NOD2

ABCG8

CNP

SLC25A19

PPARGC1A

FADS1

AKT1

HPRT1

UGT1A1

MMAB

INSR

IGF1

APOA1

GCK

ERCC5

ADSL

CRP

ARHGEF2

ACE

XDH

CYP1B1

ERCC6

GNAS

ALB

IL1B

HCN2

ABCB1

ALDH2

IL10

GSTP1

MTTP

CYP19A1

XPC

HCN1

CNGB3

ABCA1

CYP17A1

ADA

COMT

CNGA3

CFTR

HCN4

G6PD

RAPGEF3

MMUT

NOD1

RAD23B

GSTM1

GHRL

LEPR

NOS3

GCDH

NMNAT1

SECISBP2

UCP2

PAH

ERCC4

HADHA

SIRT1

CNGB1

UGT1A6

HSD11B1

ACADVL

SLC2A1

TYMS

COMMD1

BGLAP

CNGA2

GGT1

PPARD

PNP

NNT

RAPGEF1

SLCO1B1

RASGRF1

MTHFD1

CYP27B1

HIF1A

HNF4A

RETN

ARFGEF2

LMBRD1

HINT1

HMGCR

VKORC1

ARFGEF1

CES1

RAPGEF4

CD36

IRS1

SOS1

DHFR

APOA5

PON1

NAT2

PDE3B

GBF1

XPA

GNB3

CNGA1

G6PC1

PRODH

RAPGEF2

AMPD1

PCCB

PCSK9

NMNAT2

ABCD4

APOC3

EPHX1

ABCC8

ARHGEF7

LCAT

GSTT1

PC

UGT1A8

OPA3

GNA11

CPT1A

NQO1

ARHGEF1

CYP7A1

XRCC1

ARHGEF11

ACADS

PRKAG3

ARHGEF12

TCF7L2

LMNA

CCL2

GPT

ETFDH

ADK

ESR1

HCFC1

UGT1A9

RAD23A

TLR4

NMNAT3

POR

ARHGEF3

ATP7B

RAPGEF5

ERCC3

FTO

HFE

PDHA1

APRT

QDPR

PRPS1

SERPINE1

POMC

VAV1

NR3C1

SIL1

ARHGEF18

BTD

SLC19A1

SLC2A4

RAPGEF6

RALGDS

PDE5A

CPT2

AMPD3

CASR

HSD11B2

ALDH5A1

PTH

ARHGEF28

VAV3

POLG

RASGRF2

SLC25A13

FH

ARHGEF6

PRKAA1

FGF23

ADH1B

CYP24A1

ADRB3

CAT

GCG

VAV2

GSR

ARHGEF9

NR1H2

ASS1

FMO3

SLC22A5

AHCY

PRKAA2

FASN

F2

ALS2

SMPD1

DLD

UCP3

SOD1

PTGS2

AK1

CYP11B2

CNR1

DHCR7

KCNJ11

TRIO

SERGEF

BDNF

HBB

MT-ATP6

SERPINA1

CSF2RB

ARHGEF17

NR1I2

HSD17B10

SREBF1

CYP21A2

LDHA

GNAI1

RGL1

ITPA

PCCA

UCP1

PTEN

ARHGEF4

TGFB1

VEGFA

FLAD1

RANGRF

HMGCL

ADRB2

ASL

RAPGEFL1

RABGEF1

ARHGEF5

NPC1

BRCA2

RGL2

ARHGEF10

GALT

HINT2

CPS1

ARHGEF10L

SHBG

SULT1A1

MTOR

CP

DIO1

BRCA1

HMOX1

GNAI2

MAT1A

ALPL

PNPLA3

CYP27A1

REN

ARHGEF15

NT5C3A

NAMPT

ARHGEF26

UMPS

NR1H4

DDB2

SCO2

GBA1

HCN3

IVD

SDHA

FABP2

ACACA

SOD2

LIPE

TRMT10A

BCHE

BCKDHB

ETFA

PARP1

PYROXD1

ALDOB

CYP4F2

ENPP3

SLC16A1

CAD

RAC1

AGXT

HADH

RGL3

NLRP3

DDC

FAH

FBP1

UGT1A10

GSS

IMPDH2

GAA

GMPS

SOS2

RRM2B

BCS1L

SRD5A2

ARHGEF19

APOA2

TF

AK2

ERCC8

TANGO2

CYP11A1

GAPDH

OGDH

GLUD1

TFR2

ARG1

GAMT

ALOX5

TKFC

ALDH6A1

IFNG

PPIG

NT5E

NR3C2

ARHGEF40

USH2A

ABCC2

GNAO1

GK

HAMP

PYGM

PYGM
